# Supplementary material for: A Likelihood Approach for Real-Time Calibration of Stochastic Compartmental Epidemic Models
Source: PLoS Comput Biol. 2017 Jan 17;13(1):e1005257. doi: 10.1371/journal.pcbi.1005257 (PMC5240920; doi:10.1371/journal.pcbi.1005257)
Supplement: S1 File — (TAR.GZ) [file pcbi.1005257.s014.tar.gz › HSPH_Online-SI-Revision/MSS10/n100-severe/n100-severe_NEW_table-8.pdf]

1	R0	{1.65278, 9.06793, 0.1}	{1.26235, 3.50328, 0.04}	{{1.22544, 3.07157, 0.}, {1.28596, 3.98513, 0.}}	{23.6226, 61.3663, 132.}	inf	990.	414.142	{253.237, 581.167}	58.1675	0.399613	newly	963.	1043.94	{711., 1351.}	20.918	0.0873597	Reff	1.60825	1.23556	{1.19949, 1.25885}	23.1736	ar	66 342.	37 918.	{33 217., 41 349.}	42.8447
2	R0	{1.4321, 3.80644, 0.1}	{2.21785, 7.30831, 0.31}	{{1.88643, 4.56746, 0.}, {2.74056, 10.6536, 1.}}	{54.8668, 93.1376, 354.}	inf	86.	189.387	{99.2312, 272.444}	122.953	0.326887	newly	223.	352.46	{213., 511.}	61.7309	0.19692	Reff	1.42869	2.21008	{1.87949, 2.727}	54.6928	ar	54 217.	82 809.1	{75 672., 91 837.}	52.7364
3	R0	{1.68388, 4.76028, 0.1}	{1.99866, 5.99099, 0.2}	{{1.47608, 3.83841, 0.}, {2.83858, 10.1535, 1.}}	{24.9211, 39.3221, 272.}	inf	40.	68.9873	{22.0885, 121.334}	93.5512	0.297735	newly	115.	153.08	{63., 244.}	57.4261	0.223644	Reff	1.68211	1.99587	{1.47402, 2.83329}	24.8824	ar	68 299.	74 718.1	{56 111., 92 859.}	20.0939
4	R0	{1.66014, 6.95383, 1.}	{2.08315, 6.78643, 0.54}	{{1.53501, 3.37285, 0.}, {2.79531, 12.1249, 2.}}	{28.2815, 42.533, 90.}	inf	267.	328.024	{109.483, 582.63}	53.5394	0.224267	newly	379.	649.62	{443., 982.}	75.4828	0.22866	Reff	1.65028	2.06876	{1.52746, 2.7699}	28.098	ar	66 820.	77 725.7	{60 737., 92 488.}	20.2056
5	R0	{1.69984, 11.2038, 1.}	{1.25518, 3.52523, 0.11}	{{1.22544, 3.07157, 0.}, {1.28596, 3.98513, 0.}}	{26.1592, 68.5354, 95.}	inf	1644.	515.358	{368.889, 673.425}	68.6522	0.51552	newly	1210.	1266.13	{969., 1532.}	15.2686	0.0648752	Reff	1.6312	1.21863	{1.18986, 1.24873}	25.2922	ar	68 462.	37 546.7	{33 374., 41 310.}	45.1569
6	R0	{1.65876, 6.66217, 1.}	{1.33322, 3.33985, 0.06}	{{1.22544, 2.5364, 0.}, {1.47608, 4.06661, 0.}}	{21.9649, 54.9456, 102.}	inf	148.	78.6371	{11.9284, 132.556}	53.6519	0.444133	newly	251.	228.83	{49., 397.}	43.3108	0.277476	Reff	1.65251	1.32902	{1.22194, 1.47128}	21.9009	ar	67 105.	41 224.8	{32 887., 56 736.}	39.9821
7	R0	{1.69966, 5.89996, 0.1}	{2.28567, 8.88588, 0.75}	{{1.737, 4.66877, 0.}, {2.95376, 13.7076, 2.}}	{34.9468, 58.6597, 746.}	inf	95.	128.873	{53.6653, 195.004}	54.8182	0.204137	newly	161.	187.06	{113., 273.}	35.4658	0.131521	Reff	1.69633	2.28025	{1.73479, 2.94508}	34.8891	ar	69 396.	83 815.9	{70 842., 93 660.}	21.5197
8	R0	{1.53428, 6.3734, 0.1}	{2.60735, 17.4799, 3.}	{{2.47422, 16.1449, 3.}, {2.85956, 19.8999, 3.}}	{69.9395, 174.264, 2900.}	inf	389.	1337.8	{1125.29, 1630.17}	243.909	0.532095	newly	553.	736.06	{681., 800.}	33.1031	0.123073	Reff	1.51552	2.55039	{2.42064, 2.7907}	68.2851	ar	59 855.	90 331.9	{88 605., 93 131.}	50.918
9	R0	{1.58939, 3.48608, 0.1}	{1.35942, 3.90763, 0.28}	{{1.06738, 2.19254, 0.}, {1.737, 4.40955, 1.}}	{21.7831, 39.1524, 348.}	inf	10.	17.31	{3.23162, 32.3587}	103.909	0.323404	newly	29.	58.42	{11., 107.}	139.103	0.350209	Reff	1.58888	1.35886	{1.06703, 1.73628}	21.7831	ar	62 575.	36 439.1	{60., 70 497.}	48.3926
10	R0	{1.58528, 5.5593, 0.1}	{1.24408, 3.35052, 0.08}	{{1.21159, 3.07157, 0.}, {1.28596, 3.98513, 0.}}	{21.523, 39.7313, 166.}	inf	179.	95.8639	{43.5011, 149.42}	46.881	0.334557	newly	327.	256.3	{123., 394.}	32.0428	0.203863	Reff	1.57528	1.23726	{1.20576, 1.27878}	21.4578	ar	63 039.	35 617.2	{32 372., 41 395.}	43.4997
11	R0	{1.58305, 4.62721, 0.1}	{1.41926, 1.90638, 0.03}	{{1.39162, 1.89883, 0.}, {1.42206, 1.89883, 0.}}	{11.5842, 58.8006, 124.}	inf	50.	93.1539	{12.0584, 183.299}	123.38	0.398508	newly	133.	852.88	{90., 1828.}	554.376	0.712144	Reff	1.57961	1.41555	{1.38682, 1.41741}	11.6048	ar	63 222.	51 136.1	{49 195., 52 198.}	20.7024
12	R0	{1.70234, 9.34626, 0.1}	{1.2402, 3.0718, 0.}	{{1.22544, 2.5364, 0.}, {1.25474, 3.26445, 0.}}	{27.1476, 67.1334, 100.}	inf	872.	289.6	{195.142, 365.091}	66.789	0.493727	newly	836.	856.69	{581., 1098.}	18.4342	0.0826511	Reff	1.66487	1.22012	{1.20503, 1.23481}	26.7138	ar	68 213.	35 879.3	{32 889., 38 130.}	47.4011
13	R0	{1.68556, 9.74954, 1.}	{1.20708, 2.84385, 0.28}	{{1.06836, 1.81005, 0.}, {1.25474, 3.26445, 2.}}	{28.3868, 70.831, 100.}	inf	1232.	339.147	{175.355, 502.768}	72.4718	0.60777	newly	1100.	1033.85	{593., 1435.}	26.9991	0.146656	Reff	1.63051	1.1784	{1.04386, 1.22444}	27.7283	ar	68 426.	31 377.5	{13 535., 37 886.}	54.1439
14	R0	{1.59876, 9.78897, 1.}	{1.22328, 2.9896, 0.04}	{{1.22544, 2.5364, 0.}, {1.25474, 3.26445, 0.}}	{23.4859, 69.4595, 100.}	inf	1006.	323.191	{199.915, 427.187}	67.8737	0.515083	newly	885.	966.97	{564., 1314.}	26.1322	0.11038	Reff	1.55367	1.19712	{1.19759, 1.2268}	22.9491	ar	63 553.	33 952.5	{32 840., 37 168.}	46.5761
15	R0	{1.43577, 5.12819, 1.}	{1.28958, 3.94898, 0.23}	{{1.22544, 3.07157, 0.}, {1.3618, 4.98002, 1.}}	{11.9076, 32.1225, 89.}	inf	217.	157.555	{73.3157, 266.929}	40.2348	0.244166	newly	384.	364.45	{184., 521.}	32.2995	0.166441	Reff	1.42469	1.2803	{1.21643, 1.3527}	11.8346	ar	53 259.	39 977.3	{32 960., 47 997.}	27.3212
16	R0	{1.58866, 8.73191, 1.}	{1.52133, 6.86123, 0.86}	{{1.22544, 2.5364, 0.}, {2.24606, 16.668, 3.}}	{27.4115, 72.3197, 128.}	inf	604.	467.213	{117.84, 1168.8}	72.9999	0.441401	newly	587.	624.4	{376., 848.}	25.1107	0.107944	Reff	1.56477	1.49861	{1.21139, 2.20061}	26.9647	ar	64 349.	49 506.	{33 523., 85 256.}	41.2289
17	R0	{1.43012, 3.8163, 0.1}	{1.57316, 4.25051, 0.17}	{{1.24751, 2.5364, 0.}, {1.74592, 5.58477, 0.}}	{13.2151, 28.1648, 254.}	inf	64.	92.9253	{38.2507, 140.084}	64.906	0.227581	newly	190.	279.93	{108., 468.}	66.9	0.217013	Reff	1.42695	1.5691	{1.24551, 1.74185}	13.1762	ar	53 353.	59 781.4	{38 464., 71 145.}	19.8963
18	R0	{1.47965, 5.16472, 0.1}	{2.6121, 14.1023, 2.27}	{{2.47412, 12.9574, 2.}, {2.86118, 15.5722, 3.}}	{76.5349, 173.051, 2178.}	inf	233.	647.73	{543.948, 751.352}	178.144	0.435687	newly	443.	488.12	{395., 565.}	14.2393	0.0578978	Reff	1.47015	2.58426	{2.4502, 2.82884}	75.7821	ar	56 086.	90 046.6	{88 641., 93 040.}	60.551
19	R0	{1.57191, 5.2314, 0.1}	{2.12664, 9.3116, 0.92}	{{1.47488, 4.00571, 0.}, {2.86118, 15.5722, 2.}}	{39.054, 91.1042, 910.}	inf	225.	380.564	{141.365, 646.74}	88.2351	0.268517	newly	437.	442.11	{331., 545.}	16.286	0.0731135	Reff	1.563	2.11027	{1.46676, 2.83063}	38.7432	ar	63 119.	77 049.4	{55 647., 92 706.}	28.7994
20	R0	{1.54435, 5.32911, 0.1}	{1.82004, 7.36509, 0.67}	{{1.39162, 1.89883, 0.}, {2.86095, 19.9929, 2.}}	{29.3462, 110.331, 700.}	inf	191.	311.623	{62.7136, 723.726}	99.5234	0.345615	newly	372.	834.65	{242., 1900.}	141.648	0.318161	Reff	1.53601	1.80671	{1.38331, 2.82985}	29.0765	ar	61 870.	65 284.1	{49 987., 92 977.}	27.0851
21	R0	{1.57932, 4.90031, 1.}	{1.62028, 4.76996, 0.13}	{{1.39162, 3.37285, 0.}, {1.75359, 5.83416, 0.}}	{11.4998, 27.4256, 99.}	inf	57.	73.001	{21.6934, 126.959}	58.8266	0.232235	newly	102.	191.38	{65., 318.}	104.608	0.283775	Reff	1.57657	1.61712	{1.38879, 1.74969}	11.478	ar	63 595.	62 579.	{50 599., 71 505.}	14.5842
22	R0	{1.47044, 4.07694, 1.}	{1.51501, 6.17872, 0.82}	{{1.06738, 2.19254, 0.}, {2.57305, 16.884, 3.}}	{35.9352, 113.756, 126.}	inf	83.	135.396	{15.5751, 361.367}	118.612	0.410084	newly	207.	208.85	{68., 355.}	40.4783	0.221232	Reff	1.46579	1.50859	{1.0641, 2.55712}	35.7954	ar	55 895.	39 441.	{9784., 89 954.}	61.0849
23	R0	{1.64997, 3.34546, 0.1}	{1.68141, 4.61309, 0.04}	{{1.22544, 2.5364, 0.}, {2.17292, 6.28417, 0.}}	{21.3708, 46.0746, 132.}	inf	9.	23.5095	{4.45968, 45.9705}	179.76	0.406498	newly	51.	64.77	{13., 135.}	78.8824	0.345155	Reff	1.64943	1.68056	{1.22504, 2.17097}	21.3597	ar	66 404.	58 930.6	{16 952., 83 851.}	30.4443
24	R0	{1.67405, 4.50254, 0.1}	{1.9024, 4.38881, 0.15}	{{1.22544, 2.19254, 0.}, {2.67853, 6.2492, 0.}}	{31.2749, 33.4865, 232.}	inf	14.	10.9335	{2.9151, 21.4561}	52.307	0.320978	newly	40.	121.56	{7., 120.}	275.1	0.445546	Reff	1.6737	1.90203	{1.22523, 2.67813}	31.2709	ar	68 017.	60 853.8	{19., 91 548.}	37.059
25	R0	{1.50663, 3.1286, 0.1}	{2.08361, 7.13175, 0.39}	{{1.24751, 2.7937, 0.}, {2.79531, 12.3139, 2.}}	{44.1634, 132.894, 434.}	inf	3.	7.07186	{2.08112, 12.0277}	148.373	0.357942	newly	20.	28.07	{2., 42.}	123.25	0.462335	Reff	1.50652	2.08337	{1.24743, 2.79495}	44.158	ar	58 823.	67 440.8	{14., 92 477.}	48.5141
26	R0	{1.69822, 6.34992, 0.1}	{1.41097, 2.29152, 0.05}	{{1.24751, 1.89883, 0.}, {1.65456, 3.37285, 0.}}	{17.4296, 63.9126, 140.}	inf	141.	78.0883	{17.564, 137.358}	50.1343	0.38998	newly	238.	515.14	{127., 1022.}	139.504	0.357637	Reff	1.69328	1.40773	{1.24501, 1.65001}	17.3786	ar	69 816.	50 210.4	{36 534., 66 856.}	28.4936
27	R0	{1.55869, 4.92988, 0.1}	{2.48589, 12.6493, 0.46}	{{1.93419, 6.28417, 0.}, {2.93371, 17.1903, 1.}}	{59.4857, 156.584, 484.}	inf	86.	191.664	{107.272, 259.908}	123.319	0.328577	newly	214.	164.96	{123., 208.}	25.5234	0.133816	Reff	1.55537	2.47778	{1.92971, 2.92373}	59.3046	ar	62 344.	87 483.3	{77 524., 93 649.}	40.3236
28	R0	{1.46085, 6.06899, 0.1}	{1.27301, 3.23155, 0.}	{{1.22544, 2.5364, 0.}, {1.44762, 4.00571, 0.}}	{12.8773, 46.7531, 100.}	inf	547.	273.069	{154.745, 373.752}	50.0787	0.325527	newly	795.	786.84	{440., 1082.}	25.0541	0.117101	Reff	1.43891	1.25735	{1.21066, 1.42697}	12.6406	ar	55 484.	39 064.3	{33 952., 53 893.}	29.66
29	R0	{1.63017, 6.25164, 0.1}	{1.71817, 6.31199, 0.59}	{{1.24751, 2.5364, 0.}, {2.51743, 14.1978, 3.}}	{20.5812, 54.5395, 642.}	inf	248.	246.235	{107.272, 527.614}	51.3883	0.228063	newly	360.	429.97	{286., 606.}	29.6528	0.111641	Reff	1.6206	1.70746	{1.24201, 2.49512}	20.4364	ar	66 231.	63 759.3	{38 134., 89 454.}	21.3786
30	R0	{1.45173, 5.68165, 0.1}	{2.32267, 16.4727, 1.97}	{{2.35866, 16.9062, 2.}, {2.35866, 16.9062, 2.}}	{59.9932, 189.927, 1870.}	inf	352.	878.071	{829.618, 950.603}	149.452	0.393797	newly	541.	495.01	{453., 540.}	8.78189	0.0406239	Reff	1.43681	2.28646	{2.3198, 2.32279}	59.1348	ar	55 280.	86 336.5	{86 893., 87 479.}	56.1803
31	R0	{1.55808, 2.77316, 0.1}	{2.02407, 9.39461, 0.65}	{{1.28596, 4.02511, 0.}, {2.8702, 15.4074, 2.}}	{37.4668, 239.228, 656.}	inf	4.	5.61509	{2.24721, 9.27433}	65.1993	0.217917	newly	23.	17.07	{1., 18.}	107.174	0.684203	Reff	1.55797	2.02389	{1.28589, 2.86994}	37.4641	ar	60 940.	66 324.3	{12., 93 166.}	42.491
32	R0	{1.56797, 3.90327, 0.1}	{2.5223, 8.0691, 0.19}	{{1.94705, 4.66877, 0.}, {2.93906, 10.3908, 1.}}	{61.4336, 107.971, 256.}	inf	24.	43.8626	{21.6891, 69.6992}	89.3668	0.256214	newly	62.	84.24	{38., 128.}	57.5161	0.1994	Reff	1.56717	2.52048	{1.9451, 2.93748}	61.3992	ar	62 228.	87 286.8	{77 971., 93 827.}	41.2775
33	R0	{1.70027, 5.43261, 0.1}	{2.46992, 6.40652, 0.18}	{{1.88643, 4.08009, 0.}, {2.8702, 8.24535, 1.}}	{46.5167, 32.7872, 250.}	inf	66.	115.325	{53.019, 176.827}	84.886	0.256853	newly	163.	331.59	{162., 545.}	107.074	0.272967	Reff	1.69787	2.46511	{1.88286, 2.8643}	46.438	ar	68 798.	86 622.5	{75 947., 93 081.}	27.7042
34	R0	{1.61668, 6.86192, 0.1}	{1.68581, 9.4959, 0.35}	{{1.44485, 6.00596, 0.}, {1.78373, 11.799, 2.}}	{7.0574, 41.6287, 416.}	inf	325.	377.37	{251.364, 449.545}	24.0944	0.0964848	newly	465.	355.96	{300., 413.}	23.5054	0.119382	Reff	1.60218	1.66974	{1.43341, 1.76579}	6.97727	ar	64 693.	67 988.6	{54 961., 72 949.}	9.15467
35	R0	{1.55955, 3.45071, 0.1}	{2.04078, 9.09041, 0.66}	{{1.25474, 3.83841, 0.}, {2.73634, 13.7736, 2.}}	{40.4414, 165.874, 670.}	inf	11.	12.6264	{3.17667, 23.2068}	60.6732	0.280529	newly	53.	17.36	{3., 33.}	70.566	0.66487	Reff	1.55925	2.04033	{1.25454, 2.73551}	40.4351	ar	61 783.	69 621.4	{34., 92 036.}	41.686
36	R0	{1.65068, 4.91307, 0.1}	{2.33726, 10.8828, 0.27}	{{1.93419, 6.28417, 0.}, {2.64721, 13.7324, 1.}}	{43.6605, 124.344, 320.}	inf	26.	45.7495	{19.1771, 65.2932}	85.9367	0.269519	newly	80.	49.33	{23., 79.}	43.7875	0.287986	Reff	1.64972	2.33541	{1.93271, 2.64511}	43.6297	ar	66 453.	83 668.3	{77 495., 91 198.}	32.3614
37	R0	{1.46131, 3.06714, 0.1}	{1.46504, 1.96374, 0.}	{{1.39162, 1.89883, 0.}, {1.70301, 2.24898, 0.}}	{7.59927, 35.975, 100.}	inf	18.	73.9017	{18.066, 127.001}	317.237	0.568117	newly	72.	734.55	{128., 1353.}	922.958	0.889575	Reff	1.45985	1.46274	{1.38902, 1.69922}	7.61007	ar	56 452.	54 346.8	{49 782., 69 029.}	14.2686
38	R0	{1.71034, 5.2546, 0.1}	{2.2869, 8.55638, 0.48}	{{1.47488, 3.83841, 0.}, {2.93906, 13.7076, 2.}}	{41.2662, 78.1339, 516.}	inf	60.	94.1425	{23.2348, 155.733}	84.3162	0.312749	newly	144.	136.89	{57., 219.}	37.4653	0.208338	Reff	1.70807	2.28284	{1.47369, 2.93282}	41.1993	ar	69 816.	78 419.8	{38 668., 93 562.}	27.5748
39	R0	{1.43565, 5.57989, 1.}	{1.24744, 2.70146, 0.03}	{{1.24751, 2.5364, 0.}, {1.25474, 3.26445, 0.}}	{13.1099, 51.5858, 101.}	inf	400.	208.431	{103.033, 299.282}	47.994	0.330724	newly	680.	739.17	{361., 1117.}	34.8074	0.164051	Reff	1.4178	1.23432	{1.23277, 1.24097}	12.9413	ar	54 407.	36 815.6	{35 074., 38 179.}	32.333
40	R0	{1.40327, 4.00764, 0.1}	{1.33182, 2.49065, 0.01}	{{1.24751, 1.89883, 0.}, {1.47488, 3.37285, 0.}}	{7.44339, 37.9428, 108.}	inf	122.	124.85	{36.404, 231.414}	53.4457	0.294994	newly	269.	669.54	{117., 1768.}	174.758	0.400242	Reff	1.39731	1.32609	{1.24197, 1.46781}	7.43578	ar	51 976.	43 471.6	{35 665., 56 067.}	20.3683
41	R0	{1.63612, 4.26741, 0.1}	{2.54237, 3.75812, 0.02}	{{1.96007, 2.13725, 0.}, {2.99949, 5.42517, 0.}}	{55.3902, 23.1906, 116.}	inf	38.	87.9107	{18.0138, 166.975}	151.681	0.399236	newly	108.	1104.87	{264., 2096.}	928.12	0.884226	Reff	1.6346	2.53864	{1.95814, 2.99351}	55.3066	ar	65 029.	87 750.5	{78 286., 93 980.}	34.9405
42	R0	{1.57426, 6.50271, 0.1}	{2.36504, 15.5966, 1.64}	{{1.77826, 9.06807, 1.}, {2.73517, 19.6025, 2.}}	{50.9269, 140.928, 1546.}	inf	287.	721.897	{437.312, 944.884}	152.752	0.383033	newly	463.	438.62	{393., 484.}	7.64147	0.0351247	Reff	1.56141	2.33455	{1.76136, 2.69675}	50.2074	ar	62 522.	84 849.2	{72 291., 92 029.}	36.8934
43	R0	{1.37818, 5.37636, 0.1}	{1.79442, 10.4664, 1.83}	{{1.28596, 3.98513, 0.}, {2.12134, 14.6763, 3.}}	{33.0592, 106.742, 1798.}	inf	406.	878.564	{309.28, 1274.85}	125.676	0.319119	newly	728.	759.16	{679., 840.}	8.48077	0.0356618	Reff	1.35702	1.75682	{1.26616, 2.07223}	32.289	ar	50 098.	67 500.9	{40 631., 82 911.}	42.774
44	R0	{1.66606, 4.92641, 0.1}	{2.14973, 7.94563, 0.39}	{{1.53501, 4.02511, 0.}, {2.80387, 11.6897, 1.}}	{33.391, 67.7007, 428.}	inf	32.	37.6872	{9.5869, 64.7398}	51.283	0.230855	newly	79.	57.96	{22., 93.}	37.2405	0.243622	Reff	1.66501	2.14819	{1.5341, 2.80171}	33.3763	ar	67 066.	77 311.7	{57 211., 92 465.}	26.5942
45	R0	{1.623, 5.9449, 0.1}	{1.24117, 2.63642, 0.}	{{1.06738, 2.19254, 0.}, {1.25474, 3.07157, 0.}}	{23.5261, 55.6525, 100.}	inf	133.	72.2846	{12.9377, 134.655}	50.123	0.424774	newly	254.	262.36	{44., 499.}	57.4173	0.323572	Reff	1.61652	1.23696	{1.06439, 1.24965}	23.4803	ar	64 554.	34 391.2	{13 103., 39 142.}	46.7249
46	R0	{1.65235, 5.51234, 0.1}	{1.40388, 3.3975, 0.02}	{{1.24751, 2.5364, 0.}, {1.53501, 4.02511, 0.}}	{15.6094, 38.3655, 116.}	inf	70.	47.2355	{9.52751, 76.5304}	39.5139	0.305985	newly	154.	154.29	{39., 260.}	50.3701	0.281563	Reff	1.64944	1.40171	{1.24528, 1.53254}	15.5902	ar	66 509.	47 597.9	{34 587., 60 360.}	29.0169
47	R0	{1.62138, 8.82208, 0.1}	{1.73272, 8.67676, 1.2}	{{1.22544, 2.5364, 0.}, {2.46976, 17.5057, 3.}}	{34.9924, 80.3915, 1220.}	inf	1152.	1269.56	{282.032, 2647.12}	90.7941	0.443906	newly	1173.	1300.35	{942., 1598.}	21.3427	0.0884325	Reff	1.57308	1.6725	{1.19841, 2.36142}	33.7127	ar	65 598.	57 148.8	{34 271., 88 920.}	41.2471
48	R0	{1.64073, 6.69261, 0.1}	{1.48533, 4.05831, 0.01}	{{1.25474, 3.07157, 0.}, {1.737, 5.43578, 0.}}	{11.2991, 39.3613, 108.}	inf	217.	158.024	{74.5984, 239.159}	33.2625	0.203396	newly	314.	417.56	{235., 607.}	47.3503	0.176243	Reff	1.63171	1.47798	{1.24858, 1.72748}	11.2401	ar	66 438.	56 040.6	{37 936., 70 705.}	17.6881
49	R0	{1.65556, 3.796, 0.1}	{1.90643, 4.59314, 0.14}	{{1.22544, 2.5149, 0.}, {2.77593, 6.2492, 0.}}	{31.3535, 38.6298, 224.}	inf	5.	9.36246	{3.02251, 15.9134}	107.691	0.302564	newly	9.	60.79	{5., 125.}	590.556	0.572313	Reff	1.65538	1.90612	{1.22532, 2.77566}	31.3482	ar	66 575.	61 744.7	{16., 92 329.}	38.584
50	R0	{1.56828, 3.65713, 0.1}	{2.02234, 4.59107, 0.1}	{{1.44762, 2.5364, 0.}, {2.67853, 6.2492, 0.}}	{32.5005, 37.4644, 182.}	inf	37.	57.5352	{15.6343, 98.4904}	80.8672	0.286196	newly	103.	250.22	{48., 465.}	164.563	0.376228	Reff	1.56703	2.02022	{1.44668, 2.67467}	32.465	ar	62 761.	75 875.9	{55 070., 91 282.}	27.6345
